# Thinking in paracoccidioidomycosis: a delayed diagnosis of a neglected tropical disease, case report and review of clinical reports and eco-epidemiologic data from Colombia since the 2000

**DOI:** 10.1186/s12879-020-4864-8

**Published:** 2020-02-10

**Authors:** Deving Arias Ramos, John Alexander Alzate, Ángela María Giraldo Montoya, Yessica Andrea Trujillo, Leidy Yurany Arias Ramos

**Affiliations:** 1 Universidad Tecnológica de Pereira, Pereira, Colombia; 2 Grupo de Investigación en Medicina Interna, Universidad Tecnológica de Pereira, Pereira, Colombia; 3 Hospital Universitario San Jorge, Universidad Tecnológica de Pereira, Pereira, Colombia; 4 Hospital Universitario San Jorge, Pereira, Colombia

**Keywords:** Paracoccidioidomycosis, Neglected tropical disease, Colombia, Pulmonary emphysema, Oral Cancer

## Abstract

**Background:**

Paracoccidioidomycosis is a neglected tropical disease, endemic in several countries of South America including Colombia. We report a case of a patient with Chronic Multifocal Paracoccidioidomycosis with long-standing symptoms and a delayed diagnosis caused by several barriers to achieve it. We did a review of the papers published in Colombia about this disease, focusing in clinical data and eco-epidemiology with the finding of a lack of new information on this topic since the 2000 in our region.

**Case presentation:**

We present a 54-year-old man, farmer in his youth, with a chronic ulcerated lesion in the lower lip similar to a lip carcinoma, a deforming lesion in the nose, and respiratory symptoms with emphysematous lung. Lip biopsy with silver methenamine stain revealed small and large budding yeasts that resembles a “mariner’s wheel” confirming Chronic Multifocal Paracoccidioidomycosis. He was treated successfully but subsequently lost to follow up.

**Conclusions:**

It is very important to focus attention, reinforce the search and create networks for the study of neglected tropical diseases. The presented case illustrates a usual clinical presentation, but with a delayed diagnosis due to the difficulties that still occur in some regions like ours for the early recognition of a case of chronic multifocal paracoccidioidomycosis.

## Background

Paracoccidioidomycosis (PCM) is a granulomatous systemic mycosis, endemic in South America from Mexico to Argentina, with the largest number of cases reported in Brazil, Venezuela, Colombia and Argentina [1–4]. It is caused by a fungus of the genus *Paracoccidioides* that has a dimorphic behavior. It has been isolated in the nine-banded armadillo (*Dasypus novemcinctus*) [5], the naked-tailed armadillo (*Cabassus centralis*), domestic animals, cattle, horses, sheep and monkeys [3, 6, 7]. In Colombia it is not a disease of obligatory notification and therefore its incidence and prevalence is not known with certainty.

Paracoccidioidomycosis is considered a neglected tropical disease [8]. We present a case of PCM from a region of Colombia with the objective of maintaining attention to a forgotten tropical disease with a usual clinical presentation but with a delayed diagnosis. The case reviewed illustrates the difficulties still present for the diagnosis of PCM in some regions like ours and the lack of awareness in the medical community for this type of disease.

## Case presentation

**Fig. 1:**
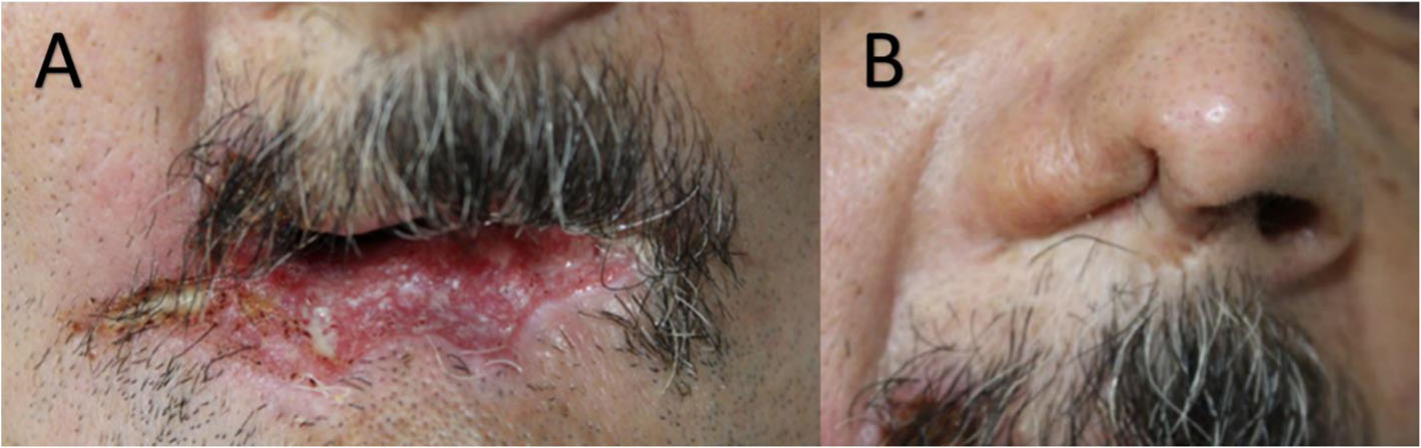
**a** Deforming and infiltrating ulcer in lower lip. **b** Deformity in the right nasal wing

A 54-year-old man from rural area of the department of Risaralda (Colombia), with a history of 3 years of a papule-like lesion in the lower lip that increased in size and then evolved to a deforming ulcer with extension towards the right labial commissure (Fig. 1). He presented a progressive deformity in the right nasal wing (Fig. 1), spontaneous loss of teeth, weight loss of 15 Kg, dry cough and dyspnea, without other B symptoms. He was a former smoker with a pack-year of 1.5 for 10 years and was receiving Metformin for a recent diagnosis of type 2 Diabetes Mellitus. He was a coffee farmer in his youth and then worked as a butcher for 30 years. Interestingly, we find that in his youth he had the habit of consuming armadillo meat and blood. He had several medical consultations, with difficulties for the follow-up caused by social aspects. A presumptive diagnosis of lip cancer was made for which a biopsy was performed.

**Fig. 2:**
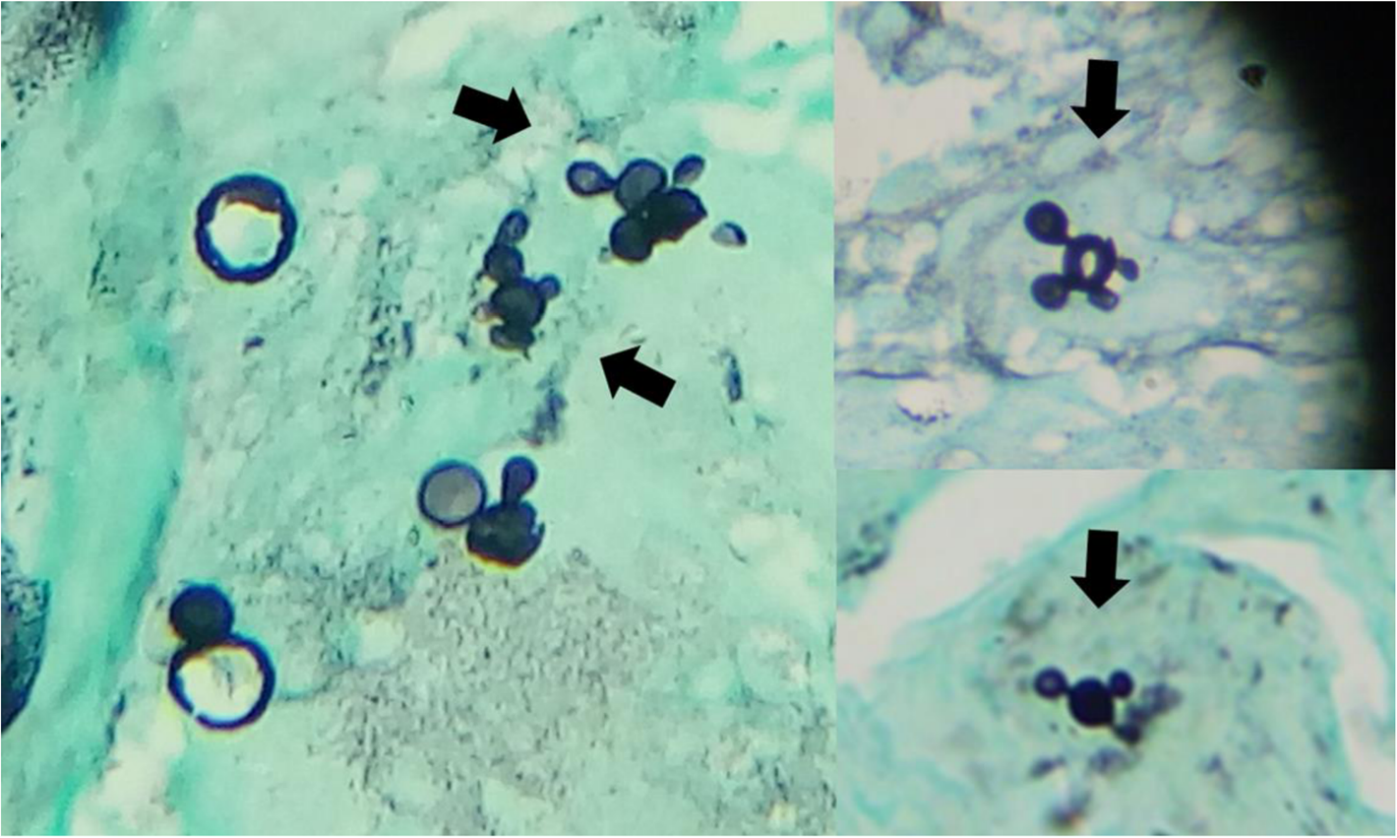
Silver methenamine stain. Budding yeasts that resembles a “mariner’s wheel”

**Fig. 3:**
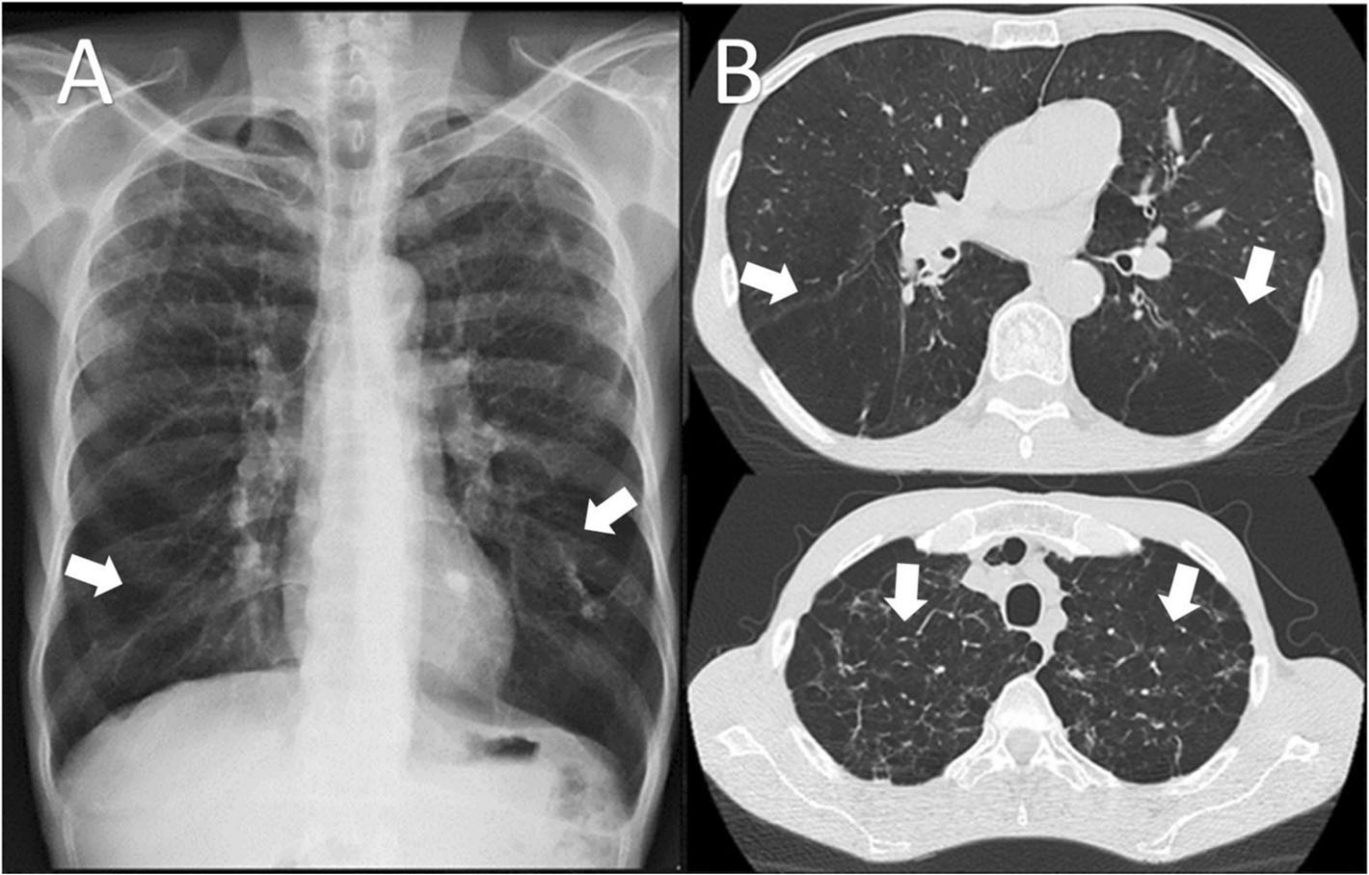
**a** Chest x-ray showing interstitial opacities and fibrotic tracts in the lung bases. **b** High Resolution Tomography of the chest. Lung window showing centrilobular and paraseptal emphysematous foci

**Fig. 4:**
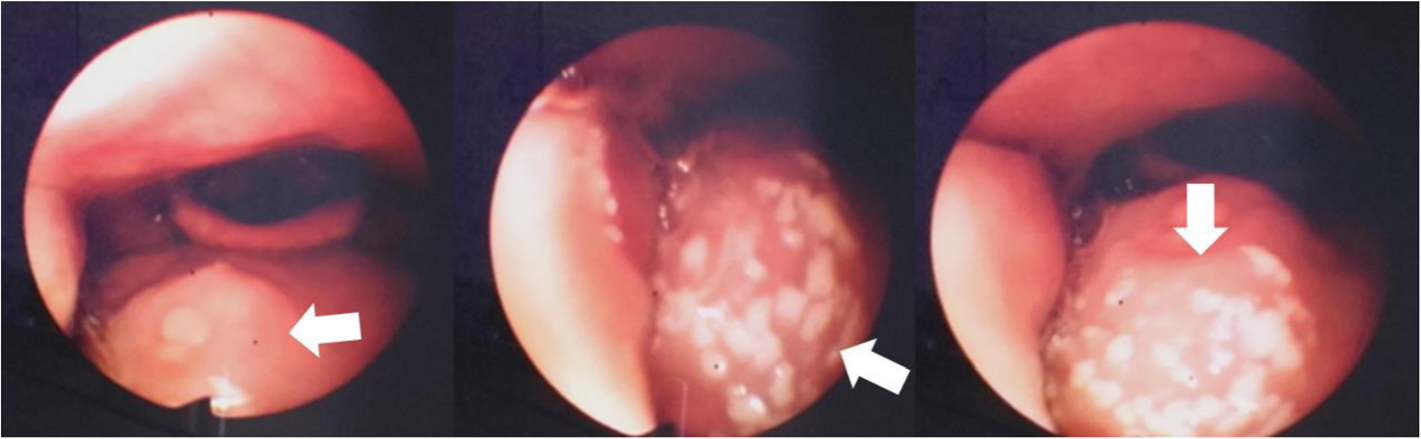
Nasofiberoptic bronchoscopy. Granulomatous lesions and ulcerated lesions covered with punctiform white secretion in upper airway

An incisional biopsy of the lip showed pseudoepitheliomatous hyperplasia associated with suppurative granulomatous dermatitis, the silver methenamine stain revealed budding yeasts that resembles a “mariner’s wheel” confirming the diagnosis of Chronic Multifocal Paracoccidioidomycosis (Fig. 2). A Chest X-ray and then a HR-CT revealed emphysematous lung disease (Fig. 3), a HIV test and a VDRL were negative. A nasofiberoptic bronchoscopy was performed to rule out tuberculosis; during the study there was seen granulomatous lesions in upper airway and other ulcerated lesions covered by punctiform white secretion (Fig. 4). There was no presence of fungal structures in the routine stains of the bronchoalveolar lavage. The culture for fungal and Mycobacterial was negative, as so the PCR for *Mycobacterium tuberculosis*.

Due to the severe constitutional commitment, he received amphotericin B for 7 days and then we made a change for Itraconazole 200 mg QD for 12 months, with successful recovery, but then lost the follow-up.

## Discussion and conclusions

Paracoccidioidomycosis can manifest in an acute/subacute form in young patients or in a chronic form mainly in adults. The chronic form is more frequent and has an age of presentation between 30 and 60 years due to a prolonged incubation period, with multifocal compromise, mostly respiratory and mucocutaneous [8–10].. Given its heterogeneous nature, this disease is considered a “great imitator” and can be a diagnostic challenge in areas of low endemicity [11, 12]. The differential diagnosis of mucocutaneous commitment includes another tropical diseases like mucocutaneous Leishmaniasis, Sporotrichosis and Histoplasmosis; other conditions like squamous cell carcinoma, non-Hodgkin’s T/Nk cell lymphoma and vasculitis should be considered [13, 14]. In the case presented, it is noteworthy that pulmonary involvement was mainly emphysematous with formation of bullae and fibrotic tracts that are associated with residual forms of this condition. The differential diagnosis is with secular forms of Tuberculosis and Histoplasmosis, and it is hardly explained by the low smoke exposure of this patient; to achieve the clinical diagnosis, the clinician can be guided if there is a concomitant presence of mucocutaneous lesions and adenopathies forming a triad of characteristic symptoms of this disease [12, 15].

Paracoccidioidomycosis is considered a neglected tropical disease [16]. Of note, in Colombia, most of the information on the eco-epidemiology of the disease comes from publication until 2000, which allowed estimating the incidence and prevalence of the disease. To mention, the prevalence is 32.4 cases/year (period 1970–1999) [17] and the incidence rate is 0.1–2.4 cases/1.000.000 inhabitants per year [18]. We also know that there is a variation in the incidence according to the geographical area, and this is caused by certain ecological characteristics such as altitudes between 1000 to 1499 m above sea level, coffee plantations and rainfall between 2000 and 2999 mm/year [1]. All these estimates have serious limitations, mainly because this disease is not for mandatory notification to the health system, so there are no reliable sources of information even in the current days.

To know about the current state of the disease in Colombia we look for publications since 2000 focusing on eco-epidemiology and clinical information. We did a search with the words “*Paracoccidioidomycosis*” “*Paracoccidioides*” and “*Colombia*” on PUBMED, BIREME (a Latin American and Caribbean Center for Information in Health Sciences), an several representative journals of Colombia: Biomédica (Journal of the National Institute of health, e-ISSN: 0120–4157), Case Reports (Journal of the Universidad Nacional, e-ISSN: 2462–8522), CES MEDICINA (Journal of Universidad CES, e-ISSN 2215–9177), Revista Médica de Risaralda (Journal of Faculty of Universidad Tecnológica de Pereira, ISSN: 2539–5203), Colombia Médica (Journal of the Universidad del Valle, ISSN-1657-9534), Medicina U.P.B (Journal of the Universidad Pontificia Bolivariana, e- ISSN: 2357–6308), IATREIA and Hechos Microbiológicos (Journals of Universidad de Antioquia, e-ISSN 2011–7965 and ISSN:2145–8898 respectively). The findings are resume in Table 1. Of note, there is no any information about possible changes in eco-epidemiology given the development of some geographic areas of the country and transition phenomena caused by the population movements from rural to urban areas for violence, and vice versa given the transition to peace since the end of the war; in addition, we do not have information on possible imported cases caused by migratory movements in recent years from Venezuela, a country endemic to this disease, and this, of course, is a challenge considering the long latent period of this disease that would force us to seek the implementation of screening tests for migrants.

**Table 1:** Papers on clinical and eco-epidemiology of paracoccidioidomycosis in Colombia since 2000

Year	Type of paper	Details of the study, Clinical and epidemiological characteristics	Ref
2000	Case report	A 24 year-old male farmer with an enlarging mass in the right testicle, retroperitoneal and inguinal nodules, pancreatic infiltration and hepatosplenomegaly due to juvenile PCM. The patient was resident of the rural area of the municipality of Tarso, department of Antioquia.	[19]
2000	Case report	A 55 year-old male with Multifocal PCM, who presented with neurological symptoms and an ulcerated granulomatous lesion in the abdomen. The patient lived in the city of Medellín, department of Antioquia, but in his younger years, was a farmer in a rural region.	[20]
2002	Case series	Six patients with adrenal insufficiency from a registry of 207 patients from the archives of the Corporación para Investigaciones Biológicas (CIB), in Medellín, department of Antioquia. Average age 67.2 years, they were residents of rural area, five of them were farmers and smokers.	[21]
2003	Case series	Follow-up for pulmonary abnormalities in 47 itraconazole-treated patients with PCM. Fibrosis was observed in 31.8% of the patients at diagnosis and developed de novo in 25% of the patients.	[22]
2005	Phylogenetic Study	Determination of at least three distinct species of PCM: S1, PS2, and PS3 (apparently restricted to regions of Colombia).	[23]
2005	Ecological study	First report of *P. brasiliensis* isolation from the spleen of a naked-tailed armadillo *Cabassous centralis* captured in a coffee farm localized in a Colombian endemic area for PCM.	[24]
2007	Case report	Autopsy diagnosis of a 63-year-old man patient, resident of the rural area of the municipality of Lebrija, department of Santander, with pulmonary, lymphatic and adrenal PCM.	[25]
2008	Retrospective cohort study	63 patients diagnosed and treated between 1978 and 2005 in the city of Medellín. 65.1% had mucosal lesions, 38.1% had Odynophagia and dysphagia, all patients had lung interstitial infiltrates and fibrosis was recorded in 46%.	[26]
2009	2 cases	Report of two cases of adrenal insufficiency secondary to infiltration of the adrenal glands. The case 2 lived in the city of Medellín, but with a history of previous residence in Venezuela	[27]
2010	Cross-sectional study: 28 patients.	Evaluation of the status of the adrenal gland function after completion of antifungal therapy of patients with PCM who had been treated earlier at the CIB: 7.1% showed adrenal insufficiency. Average age 55.3 years, all were male patients, 39.3% were farmers and 85.7% were smokers, 60.7% had chronic multifocal form, 21.4% had sub-acute form, and 17.9% had the chronic unifocal form.	[28]
2010	Case report	A 31-year-old HIV infected male patient, *Embera Catío* indigenous, resident of rural area of the municipality of Tarazá (department of Antioquia), works as a coca leaf scraper and with a history of smoking; presenting with simultaneous co-infection by *Histoplasma capsulatum* and *Paracoccidioides brasiliensis.*	[29]
2011	Case report	56-year-old male with Multifocal chronic PCM, residing in an urban area of the city of Cali (department of Valle del Cauca); he had emigrated from a wooded region of the city of Pasto (department of Nariño) where he performed agricultural and carpentry tasks.	[30]
2011	Case series	A descriptive case series study aimed at determining the type and frequency of opportunistic diseases in HIV/AIDS patients: 1 case with co-infection by *P. brasiliensis*.	[31]
2014	Case report	A 12-year-old woman with disseminated juvenile PCM diagnosed in an urban area of Bogotá, Colombia.	[32]
2015	Case report	A 69-year-old HIV infected male patient with a CD4 T-cell count of 23 cells/mL, presenting with Cutaneous PCM. He was a farmer in a rural area of the department of Antioquia.	[33]
2016	Case report	A 10-year-old man from the rural area of the municipality of Yotoco, department of Valle del Cauca, presenting with subacute lymphatic PCM.	[34]
2017	Case report	67-year-old male with pulmonary PCM, resident of the rural area of the municipality of Aratoca, department of Santander, worked as a farmer, with a history of smoking.	[35]
2018	Case report	49-year-old male, coffee grower, from City of Acevedo, department of Huila, with a history of smoking, presenting with Multifocal chronic PCM.	[36]

*PCM* paracoccidioidomycosis, *CIB* Corporación para Investigaciones Biológicas

The lack of new information in the last two decades on the eco-epidemiology and the clinical and social characterization of patients with Paracoccidioidomycosis reaffirms it as a neglected tropical disease, an aggravated situation during the last two decades despite the era of informatics, and also it is a call for physicians, institutions, and government to start working on this issue again. The main problems to recognize PCM seem to be 1) the apparent rare occurrence of the disease; 2) the difficulty to diagnose the disease in a timely manner; 3) the lack of interest of the clinicians to make it more visible, from the instances of the health system to the instances of medical education at the individual level. From this perspective, it seems that the way to begin to solve this problem could be to create a national network that involves leaders in the different medical schools.

## Data Availability

The datasets used and/or analysed during the current study available from the corresponding author on reasonable request.

## Abbreviations

BAL: Bronchoalveolar lavage
BMI: Body mass index
HIV: Human Immunodeficiency Virus
HR-CT: High-resolution Computed Tomography
Kg: Kilogram
PCM: Paracoccidioidomycosis
PCR: Polymerase chain reaction
QD: “quaque die”, which means, in Latin, once a day
T2DM: Type 2 Diabetes Mellitus
VDRL: Venereal Disease Research Laboratory
